# Spatial and temporal analysis of viral hepatitis in mainland China from 2007 to 2023: implications for targeted public health interventions

**DOI:** 10.3389/fpubh.2025.1660415

**Published:** 2025-12-04

**Authors:** Qiang Liu, Yi Fei Li

**Affiliations:** 1 College of Resources and Environmental Engineering, Tianshui Normal University, Tianshui, China; 2 State Key Laboratory of Soil Erosion and Dryland Farming on the Loess Plateau, Institute of Soil and Water Conservation, Chinese Academy of Sciences, Ministry of Water Resources, Yangling, China

**Keywords:** viral hepatitis, spatial epidemiology, China, public health surveillance, spatiotemporal analysis, disease mapping

## Abstract

**Background:**

Viral hepatitis remains a major public health challenge in China, contributing significantly to global disease burden. Understanding spatiotemporal patterns is crucial for developing effective control strategies.

**Methods:**

We conducted spatiotemporal analysis of viral hepatitis surveillance data from 31 provinces in mainland China spanning 2007–2023, encompassing 21,346,298 reported cases of hepatitis A, B, C, and E. Methods included descriptive epidemiological analysis, spatial autocorrelation using Moran’s *I* statistics, and spatial correlation analysis with transportation networks and water systems using Geographic Information Systems (GIS).

**Results:**

Hepatitis B dominated with 78.63% of cases, followed by hepatitis C at 12.51%. The annual incidence of hepatitis A decreased from 7.20 to 1.06 (85.3% reduction, APC = −15.2%); hepatitis C increased from 3.03 to 13.82 (356% growth, APC = +11.8%); hepatitis B showed slight decline (APC = −0.6%); and hepatitis E remained relatively stable (APC = +1.4%). Spatial autocorrelation analysis revealed significant clustering patterns (Global Moran’s *I*: 0.412 for hepatitis A, 0.387 for hepatitis B, 0.523 for hepatitis C, and 0.298 for hepatitis E; *p* < 0.01), with persistent high-incidence clusters in western provinces (Xinjiang, Tibet, and Qinghai) for hepatitis A and C, while hepatitis E showed significant clustering in eastern coastal regions (Zhejiang, Jiangsu, and Shandong). Infrastructure analysis demonstrated significant negative correlations between transportation proximity and incidence (*p* < 0.001).

**Conclusion:**

Our findings validate hepatitis A vaccination as a cost-effective elimination strategy that can be replicated in resource-limited settings; identify the need for immediate policy action on hepatitis C; and demonstrate that investment in transportation infrastructure can reduce hepatitis burden by improving healthcare access in remote areas. These studies provide theoretical references for China to achieve the 2030 elimination target and offer transferable strategies for countries facing similar geographical and socio-economic challenges.

## 1 Introduction

Viral hepatitis constitutes one of the most significant infectious disease challenges globally, with an estimated 1.5 million deaths annually and affecting over 325 million people worldwide (1). The global economic burden exceeds $24 billion annually, including direct medical costs, productivity losses, premature mortality, and long-term care requirements for patients with liver disease (2). The disease burden varies significantly across different hepatitis types, with hepatitis B and C responsible for the majority of chronic infections and liver-related deaths globally, while hepatitis A and E predominantly cause acute infections with potential for epidemic outbreaks (3).

Recognizing this global health crisis, the World Health Organization established an ambitious Global Health Strategy in 2016, targeting complete elimination of viral hepatitis as a public health threat by 2030 through a multifaceted framework aiming for 90% reduction in new infections and 65% reduction in mortality compared to 2015 baseline levels (4). This elimination strategy represents one of the most ambitious infectious disease control initiatives in modern public health history, requiring coordinated global action, substantial resource mobilization, innovative intervention approaches, and sustained political commitment across all affected countries (5).

The global epidemiology of viral hepatitis demonstrates geographic heterogeneity, with distinct transmission patterns and disease burdens across different world regions. High-income countries have generally achieved substantial progress through systematic vaccination programs, improved blood safety measures, and effective harm reduction strategies, while low- and middle-income countries continue to bear disproportionate disease burdens, facing challenges including limited healthcare infrastructure, inadequate surveillance systems, and insufficient access to prevention and treatment services (6).

China plays a central role in global viral hepatitis control, bearing the heaviest worldwide disease burden with approximately 70 million chronic hepatitis B infections and 10 million chronic hepatitis C infections, representing nearly one-third of the entire global hepatitis burden and making China’s progress essential for achieving global elimination goals (7, 8). This high disease load reflects a complex interplay of historical factors including decades of inadequate blood product screening during the 1980s and 1990s, suboptimal infection control practices in healthcare settings, traditional medical practices involving blood contact, widespread injection drug use in certain regions, and insufficient vaccination coverage in earlier decades (9, 10).

The demographic transition occurring in China, characterized by rapid aging of the population, presents unique challenges for hepatitis management as chronic infections acquired decades earlier progress to liver disease in growing numbers of older individuals. Concurrently, massive internal migration patterns affecting over 280 million people have facilitated disease transmission across geographic boundaries while creating challenges for continuity of care and surveillance systems (11). China’s unique position as the world’s most populous country with the highest absolute disease burden makes its elimination progress critical for global targets, with success or failure potentially determining worldwide elimination feasibility.

The epidemiological landscape of viral hepatitis in China exhibits heterogeneity across multiple interconnected dimensions, reflecting the country’s vast geographic diversity spanning tropical to arctic climates, significant socioeconomic disparities between developed coastal regions and underdeveloped interior areas, varied healthcare infrastructure development ranging from world-class urban medical centers to basic rural clinics, and complex demographic transitions including rapid urbanization, population aging, and massive internal migration patterns (11, 12). This multidimensional heterogeneity manifests in distinct and persistent regional patterns, with western provinces consistently experiencing disproportionately higher disease burdens compared to eastern coastal regions, pronounced urban–rural disparities in access to prevention and treatment services, significant variations in healthcare quality and availability, and complex demographic transitions affecting age-specific annual incidence patterns across different hepatitis types and geographic areas (13).

Urban–rural disparities represent another critical dimension of hepatitis epidemiology in China, with rural populations typically experiencing higher hepatitis prevalence, reduced access to prevention and treatment services, and significant delays in diagnosis and care initiation. These disparities reflect broader healthcare system inequities including uneven distribution of healthcare resources, provider shortages in rural areas, and financial barriers to care access that disproportionately affect rural and economically disadvantaged populations.

The four major types of viral hepatitis A, B, C and E, exhibit distinct transmission pathways, clinical manifestations, natural histories, geographic distribution patterns, and prevention requirements, necessitating carefully tailored and type-specific prevention and control approaches (14). Hepatitis A virus and hepatitis E virus are primarily transmitted through the fecal-oral route, often associated with poor sanitation conditions, contaminated water sources, inadequate food safety practices, overcrowded living conditions, and insufficient personal hygiene practices (15, 16). These enteric hepatitis types typically cause acute infections with significant potential for explosive epidemic outbreaks, particularly in areas with suboptimal sanitation infrastructure, vulnerable populations, and inadequate public health preparedness.

In stark contrast, hepatitis B virus and hepatitis C virus are blood-borne pathogens transmitted through direct exposure to infected blood and body fluids, with important implications for vertical transmission from infected mothers to newborns, healthcare-associated infections in medical settings, injection drug use among high-risk populations, unsafe medical and cosmetic procedures, and other high-risk behaviors involving blood contact or sharing of contaminated instrument (17, 18). The complex natural history of chronic hepatitis B and C, characterized by decades-long infection periods with potential progression to cirrhosis and hepatocellular carcinoma, creates substantial long-term healthcare burdens and requires approaches addressing both prevention of new infections and management of existing chronic infections through effective antiviral therapies and regular monitoring for disease progression.

Recent decades have witnessed changes in China’s viral hepatitis epidemiology, driven by sustained public health interventions, socioeconomic development, massive healthcare system strengthening initiatives, and fundamental changes in population behavior and risk factors (19). The implementation of systematic national vaccination programs represents one of the most significant and successful public health achievements in modern Chinese history, particularly the strategic integration of hepatitis A vaccine into the nationally coordinated Expanded Program on Immunization in 2008 and the progressive expansion of universal hepatitis B vaccination coverage since the early 1990s, which has fundamentally altered disease transmission patterns and population susceptibility (10, 20).

These vaccination programs have created and sustained reductions in vaccine-preventable hepatitis types while simultaneously creating new epidemiological challenges related to aging infected populations, potential waning immunity in vaccinated cohorts, and shifting disease burden to previously low-risk age groups. The success of these programs has been facilitated by China’s robust and expanding primary healthcare infrastructure, strong governmental commitment to vaccination initiatives, effective integration with existing childhood immunization services, and improvements in vaccine delivery systems reaching even the most remote rural areas.

Concurrent with systematic vaccination program implementation, China has experienced rapid economic development, massive urbanization affecting hundreds of millions of people, improved living standards across all socioeconomic strata, enhanced sanitation infrastructure including modern water treatment and waste management systems, and strengthening of healthcare systems at all levels from community clinics to specialized hepatology centers. However, these developments have been accompanied by emerging challenges including increased population mobility facilitating disease spread, changing lifestyle patterns affecting risk behaviors, and growing health inequities between developed coastal regions and underdeveloped interior areas.

Spatial epidemiology has emerged as an increasingly powerful and scientific discipline for understanding complex disease distribution patterns, identifying previously unrecognized risk factors and transmission pathways, and informing evidence-based public health interventions that can achieve maximum impact with available resources (21, 22). The integration of Geographic Information Systems technology with spatial statistical methods enables analysis of disease clustering patterns, precise identification of high-risk geographic areas requiring targeted interventions, examination of relationships with environmental and infrastructure factors, and development of predictive models for enhanced disease surveillance and proactive control measures.

Contemporary spatial epidemiological research has demonstrated value and practical utility for infectious disease control efforts, enabling identification of previously unrecognized disease clusters, characterization of transmission hotspots and risk factors, rigorous evaluation of intervention effectiveness and impact, and strategic optimization of resource allocation to achieve maximum public health benefit (23). For viral hepatitis specifically, spatial analysis approaches can reveal important patterns including geographic clustering of high-annual incidence areas requiring immediate attention, complex relationships between disease distribution and transportation networks affecting healthcare access, associations with water systems and environmental factors influencing transmission risk, and precise identification of populations and geographic areas requiring urgent targeted interventions.

Previous research examining viral hepatitis epidemiology in China has primarily focused on national-level surveillance data analysis providing broad overview perspectives, regional studies of specific hepatitis types offering limited geographic scope, or limited-duration trend analyses lacking sufficient temporal depth, often lacking the spatiotemporal perspective necessary for understanding complex disease patterns and informing effective strategic interventions (24, 25). While these valuable studies have provided important insights into specific aspects of China’s viral hepatitis burden and contributed to our understanding of disease patterns, analyses covering multiple hepatitis types over extended periods with spatial analytical approaches remain limited and insufficient for guiding elimination efforts.

The complexity and scale of China’s viral hepatitis challenge requires innovative and analytical approaches that can effectively capture the multidimensional nature of disease patterns while providing actionable and practical insights for policy development and program implementation at national, provincial, and local levels. The strategic integration of long-term surveillance data with spatial analytical methods offers opportunities to understand intricate disease patterns, identify optimal intervention targets, evaluate progress toward ambitious elimination goals, and develop evidence-based strategies for achieving sustainable disease control.

This study addresses these critical knowledge gaps by conducting the most extensive and spatiotemporal analysis of viral hepatitis in mainland China undertaken to date, covering an 17-year period from 2007 to 2023 and encompassing over 21 million reported cases across all 31 provinces, autonomous regions, and municipalities. Our primary research objectives include: characterizing temporal trends and demographic patterns for hepatitis A, B, C, and E to understand long-term epidemiological transitions and their underlying determinants; systematically identifying spatial clustering patterns and clustering using spatial statistical methods to inform targeted intervention strategies; rigorously examining complex relationships between disease distribution and geographic factors including transportation infrastructure and water systems that may influence transmission patterns; objectively evaluating progress toward World Health Organization (WHO) elimination targets and identifying persistent challenges requiring immediate attention; and providing evidence-based recommendations for targeted public health interventions and policy development to accelerate elimination efforts.

## 2 Materials and methods

### 2.1 Study area

**Figure 1:**
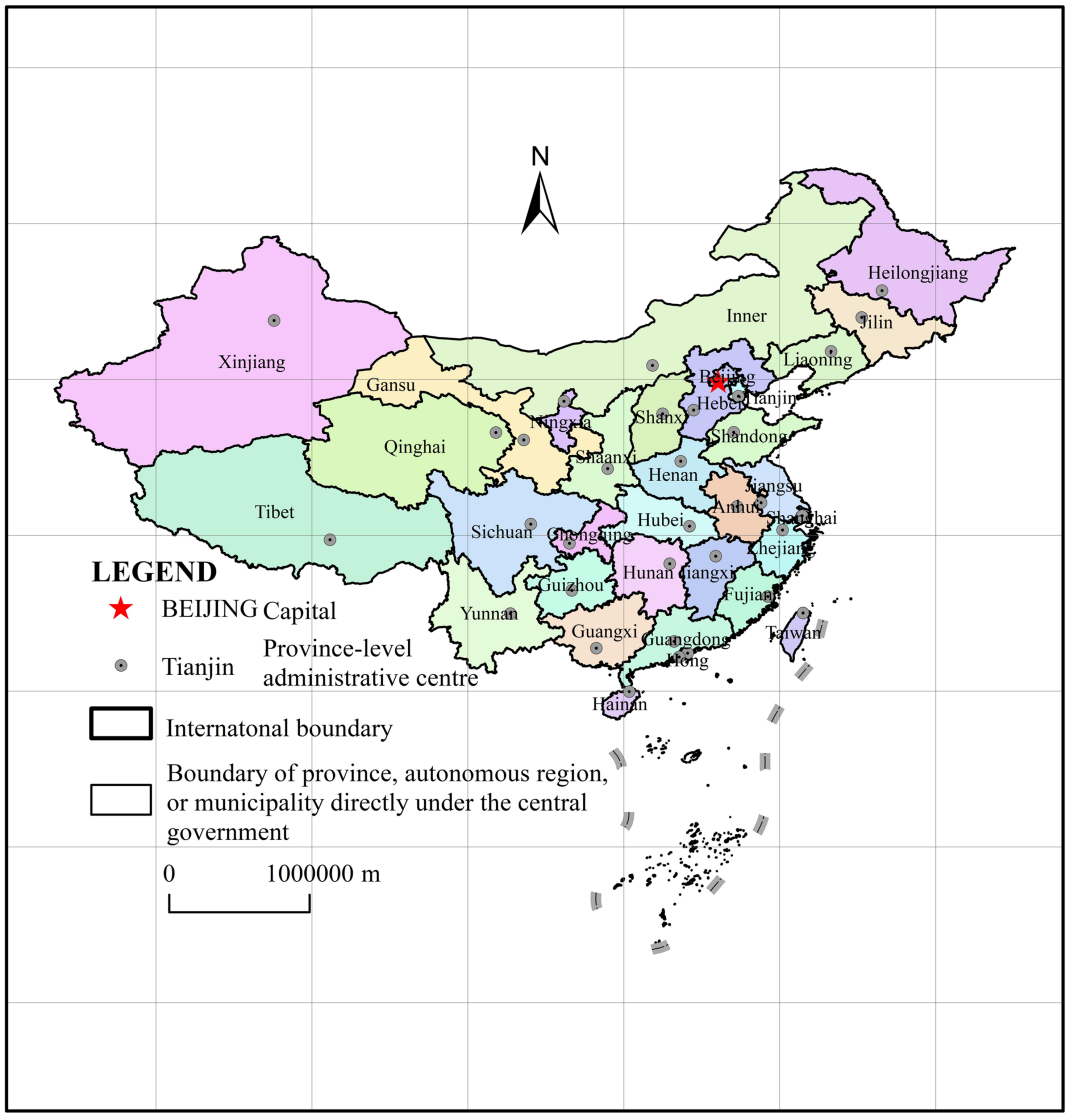
Map of mainland China.

This study encompasses all 31 provincial-level administrative divisions in mainland China, covering approximately 9.6 million km^2^ (Figure 1). The study area comprises 22 provinces (Hebei, Shanxi, Liaoning, Jilin, Heilongjiang, Jiangsu, Zhejiang, Anhui, Fujian, Jiangxi, Shandong, Henan, Hubei, Hunan, Guangdong, Hainan, Sichuan, Guizhou, Yunnan, Shaanxi, Gansu, and Qinghai), 5 autonomous regions (Inner Mongolia, Guangxi, Tibet, Ningxia, and Xinjiang), and 4 municipalities directly under central administration (Beijing, Tianjin, Shanghai, and Chongqing). China’s vast territory spans diverse geographic zones from 18 °N to 54 °N latitude and 73 °E to 135 °E longitude. The topography is characterized by three major terraces descending from west to east: the Tibetan Plateau with average elevation exceeding 4,000 m, the central plateaus and basins ranging from 1,000 to 2,000 m, and the eastern coastal plains below 200 m. Climate zones range from tropical monsoon in southern Hainan to temperate continental in northern Heilongjiang, with the southeastern regions experiencing humid subtropical conditions while northwestern areas are predominantly arid and semi-arid.

### 2.2 Study design and setting

We implemented a retrospective ecological study design specifically optimized to analyze viral hepatitis surveillance data from all 31 provinces, autonomous regions, and municipalities in mainland China over an extensive 17-year period from 2007 to 2023. This analytical approach was strategically selected to capture long-term epidemiological trends while enabling spatial analysis of complex disease patterns across China’s extraordinarily diverse geographic, socioeconomic, demographic, and healthcare landscape (11). This design balances temporal depth with spatial resolution to effectively identify patterns and inform targeted interventions.

The study encompassed four major viral hepatitis types with sufficient surveillance data availability and public health significance: hepatitis A, B, C, and E, which collectively represent the overwhelming majority of China’s viral hepatitis burden and provide coverage of both enteric and blood-borne hepatitis transmission patterns (14).

The temporal scope of 2007–2023 was strategically selected to capture several key periods and transition points in China’s viral hepatitis control efforts and broader healthcare development, including the important integration of hepatitis A vaccine into the national immunization program in 2008, progressive expansion of hepatitis B treatment programs using modern antiviral therapies, implementation of enhanced surveillance systems with improved case detection capabilities, recent accelerated progress toward WHO elimination targets, and the challenges posed by the COVID-19 pandemic during 2020–2023 (19). This study encompasses all 31 provincial-level administrative divisions of mainland China, including 22 provinces, 5 autonomous regions, and 4 municipalities. All territories, including island provinces and regions, are included in both the epidemiological surveillance data and spatial analysis.

### 2.3 Data sources and collection

#### 2.3.1 Epidemiological surveillance data

Viral hepatitis surveillance data were systematically obtained from the Public Health Science Data Center of the Chinese Center for Disease Control and Prevention, representing the most authoritative and reliable source of viral hepatitis surveillance data in China and operating under strict quality assurance protocols (26). The national surveillance system operates through a highly organized hierarchical network extending systematically from national headquarters to provincial centers, prefecture-level offices, county-level facilities, and township-level reporting units, ensuring geographic coverage, standardized data collection procedures, and consistent reporting protocols across all administrative levels. The surveillance system captures all laboratory-confirmed viral hepatitis cases reported through the nationally integrated notifiable disease surveillance network, operating under rigorously enforced protocols established by the National Health Commission of China in collaboration with international organizations including WHO and CDC (27). Case ascertainment follows the standardized case definitions established by the World Health Organization (WHO, 2017) and adapted by the Chinese Center for Disease Control and Prevention (China CDC, 2019), which require laboratory confirmation through specific serological markers: anti-HAV IgM for hepatitis A, HBsAg and/or anti-HBc IgM for hepatitis B, anti-HCV with confirmatory RNA testing for hepatitis C, and anti-HEV IgM for hepatitis.

Surveillance data included annual case counts systematically stratified by province, age group categories, sex, and specific hepatitis type, along with corresponding population-based annual incidence calculated using official population estimates from the National Bureau of Statistics with appropriate adjustments for demographic transitions and census updates (28).

Annual incidence were calculated using the formula: annual incidence = (Number of new cases per year/Mid-year population) × 100,000. Denominator population data were obtained from annual census and sample survey data published by the National Bureau of Statistics, using mid-year population (July 1) as the exposure population base. For provincial annual incidence, resident population data for corresponding provinces and years were used. Annual incidence are expressed population and rounded to two decimal places. Age-specific annual incidence used corresponding age group populations as denominators, and sex-specific rates were calculated using respective sex populations.

Concerning the potential for duplicate records from the same individuals, it should be noted that healthcare institutions are mandated to report all new cases, including both incident and relapse cases of the same patient. Consequently, the possibility exists for repeated reporting of the same individual.

#### 2.3.2 Geographic and infrastructure data

High-resolution administrative boundary maps and precise coordinate reference systems were systematically obtained from the National Geomatics Center of China, providing official provincial boundaries, accurate geographic reference frameworks, and standardized cartographic foundations essential for spatial analysis and reliable geographic information system applications (29).

Transportation network data, including information on railway systems, highway networks, and major transportation hubs, were systematically compiled from authoritative OpenStreetMap contributors and supplemented with official transportation ministry datasets to ensure complete coverage, accuracy, and temporal consistency (30).

Hydrological data encompassing major river systems, lakes, reservoirs, watershed boundaries defined by topographic drainage divides, and water quality monitoring stations were compiled from the Ministry of Water Resources (31), China Institute of Water Resources and Hydropower Research (IWHR, 2020), and the National Earth System Science Data Center (NESSDC, 2021), supporting comprehensive environmental analysis and understanding of water-related transmission pathways (31–33).

#### 2.3.3 Socioeconomic and healthcare infrastructure data

Temporal trend analysis employed multiple statistical approaches specifically designed to characterize long-term patterns, identify significant trend changes with maximum statistical power and precision, and distinguish genuine epidemiological transitions from surveillance artifacts. Linear regression analysis was systematically used to quantify overall trends across the entire study period, calculating average annual percent change with 95% confidence intervals for each hepatitis type and province using established methods (32). Based on the results of Joinpoint regression analysis, temporal trends were classified according to the following criteria: (1) Increasing trend: Annual Percent Change (APC) > 0 with *p* < 0.05; (2) Decreasing trend: APC < 0 with *p* < 0.05; (3) Stable trend: *p* ≥ 0.05 regardless of APC value, indicating no statistically significant change. For magnitude assessment, we further categorized significant trends as: marked increase/decrease (|APC| > 10%), moderate increase/decrease (5% ≤ |APC| ≤ 10%), and slight increase/decrease (|APC| < 5%). These standardized criteria ensure consistent and reproducible trend classification throughout the analysis (33).

Global spatial autocorrelation analysis employed Moran’s *I* statistic to quantify overall spatial clustering of hepatitis annual incidence across provinces for each year and hepatitis type, providing rigorous statistical assessment of spatial pattern significance (34). The Moran’s *I* statistic was calculated using established formulas with statistical significance assessed through standardized z-scores and Monte Carlo simulation procedures. Local indicators of spatial autocorrelation analysis systematically identified specific spatial clustering patterns and outlier provinces requiring investigation and potential targeted interventions (35). The analysis generates four distinct categories of local spatial association: High-High clusters, Low-Low clusters, High-Low outliers, and Low-High outliers (36).

Buffer analysis examined complex relationships between transportation networks, water systems, and hepatitis annual incidence patterns using three complementary analytical approaches: distance-decay analysis, kernel density estimation, and spatial overlay analysis. Buffer zones were systematically created at multiple carefully selected distance intervals including 10, 20, 50, 100, and 200 km to capture varying scales of potential influence.

### 2.4 Spatial autocorrelation analysis

This study employed global and local Moran’s *I* statistics to assess spatial clustering patterns of viral hepatitis. The spatial weight matrix was constructed based on Queen contiguity criterion, where provinces sharing any boundary or vertex were defined as neighbors, with island provinces such as Hainan having neighbors defined by shortest maritime distance (<100 km), and the matrix was row-standardized to ensure comparability. Considering Moran’s *I* sensitivity to data non-stationarity, we applied logarithmic transformation to stabilize variance in raw rates and performed detrending to remove long-term temporal trends. Statistical significance of spatial autocorrelation (Moran’s *I* statistic) was assessed through Monte Carlo permutation tests with 9,999 permutations, where observed values were randomly reassigned among spatial units in each permutation to generate the reference distribution under the null hypothesis of spatial randomness, adopting hierarchical significance criteria of *p* < 0.05 for significant, *p* < 0.01 for highly significant, and *p* < 0.001 for extremely significant spatial clustering. Given the data constraints that hindered age standardization, we adopted alternative analytical strategies. These included within-province temporal comparisons, relative ranking analyses, and various descriptive statistical methods alongside regression techniques. Specifically, we employed Moran’s *I* spatial autocorrelation analysis, Joinpoint regression, linear regression, and polynomial regression to mitigate the effects of demographic heterogeneity on our conclusions.

Statistical and spatial analyses were performed using the following software: R software (Version 4.2.0, R Foundation for Statistical Computing, Vienna, Austria) for descriptive statistical analysis and regression analysis; GeoDa software (Version 1.20, University of Chicago, Chicago, IL, USA) for spatial autocorrelation analysis; ArcGIS software (Version 10.8, Esri Inc., Redlands, CA, USA) for spatial data visualization and geographic information system analysis; Joinpoint Regression Program (Version 5.0.2, National Cancer Institute, Bethesda, MD, USA) for temporal trend analysis. All statistical tests employed two-tailed tests with significance level set at *α* = 0.05.

## 3 Results

### 3.1 Overall disease epidemiological characteristics

During 2007–2023, mainland China reported a total of 21,346,298 viral hepatitis cases. Joinpoint regression analysis revealed distinctly different epidemiological trajectories and critical turning points for the four hepatitis types (Table 1). Hepatitis A demonstrated the most declining trend with an Average Annual Percent Change (AAPC) of −15.2% (95% CI: −16.8% to −13.5%, *p* < 0.001). Annual incidence plummeted from 7.20 in 2007 to 1.06 in 2023, representing an 85.3% reduction. Joinpoint analysis identified two critical turning points in 2008 and 2015: following hepatitis A vaccine integration into the national immunization program in 2008, rapid decline was sustained during 2008–2015 (APC = −18.4%); deceleration after 2015 (APC = −6.2%) suggests approaching elimination levels.

**Table 1:** Joinpoint regression analysis of viral hepatitis trends in mainland China, 2007–2023.

Hepatitis type	Period	APC (%)	95% CI	*p*-value	AAPC (2007–2023)	95% CI	*p*-value
Hepatitis A	2007–2008	−21.6	−28.4 to −14.2	<0.001	−15.2	−16.8 to −13.6	<0.001
	2008–2015	−18.4	−20.1 to −16.6	<0.001			
	2015–2023	−6.2	−8.5 to −3.8	<0.001			
Hepatitis B	2007–2010	+8.7	+6.2 to +11.3	<0.001	−0.6	−1.2 to 0.0	0.052
	2010–2023	−3.2	−4.1 to −2.3	<0.001			
Hepatitis C	2007–2012	+22.6	+19.8 to +25.5	<0.001	+11.8	+10.2 to +13.4	<0.001
	2012–2023	+4.8	+3.9 to +5.7	<0.001			
Hepatitis E	2007–2023	+1.4	−0.3 to +3.1	0.102	+1.4	−0.3 to +3.1	0.102

Hepatitis B, constituting the primary disease burden (78.63% of total cases), showed marginally significant declining trends (AAPC = −0.6, 95% CI: −1.2 to 0.0%, *p* = 0.052). The year 2010 marked a critical turning point, with increasing trends before (APC = +8.7%) likely reflecting improved surveillance, transitioning to sustained decline during 2010–2023 (APC = −3.2%, *p* < 0.001), manifesting cumulative effects of neonatal vaccination programs. Despite substantial absolute case numbers (averaging ~1 million annually), established declining trends provide foundation for achieving 2030 elimination targets.

Hepatitis C exhibited the most concerning epidemic trajectory with an AAPC of +11.8% (95% CI: +10.2% to +13.4%, *p* < 0.001), annual incidence surging from 3.03 in 2007 to 13.82 in 2023, a 356% increase. The year 2012 represented a turning point, with rapid growth before (APC = +22.0%) and decelerated but sustained increases after (APC = +4.8%). This persistent upward trend seriously threatens WHO 2030 elimination goals, urgently requiring intensified control measures. Hepatitis E maintained relatively stable endemic levels (AAPC = +1.4%, 95% CI: −0.3% to +3.1%, *p* = 0.102), averaging 1.68 annually without significant temporal trends.

### 3.2 Geographic distribution analysis

**Figure 2:**
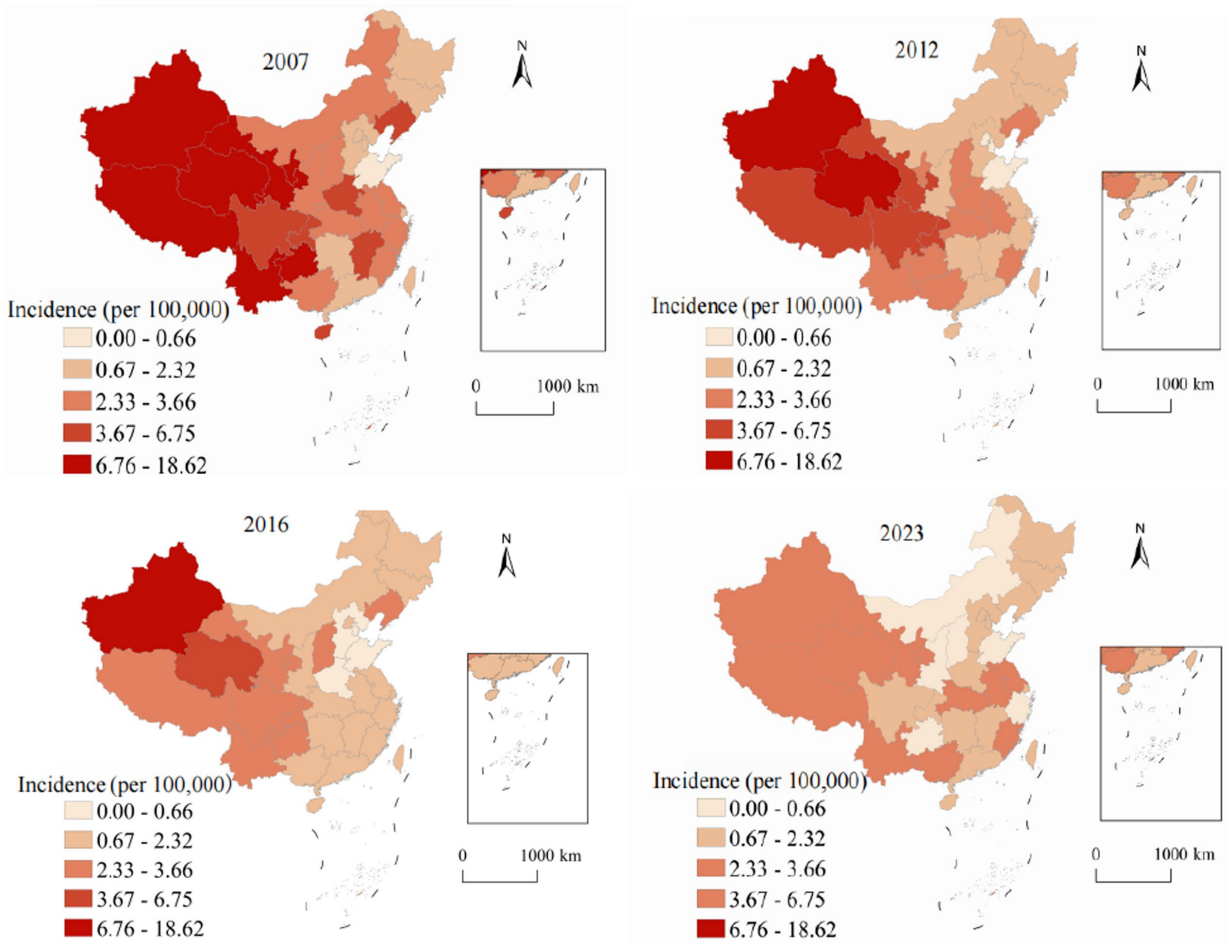
Analysis of the annual incidence distribution of hepatitis A in China from 2007 to 2023.

Spatial analysis revealed pronounced and persistent geographic variation in viral hepatitis distribution across China’s 31 provinces, autonomous regions, and municipalities, reflecting complex interactions between environmental factors, socioeconomic conditions, healthcare infrastructure development, and demographic characteristics. Figure 2 demonstrates spatial and temporal evolution of hepatitis A annual incidence from 2007 to 2023. In 2007, pronounced west–east disparities were evident, with western provinces showing the highest rates: Xinjiang at 28.4, Tibet at 22.1, and Qinghai at 18.7, while eastern coastal provinces maintained substantially lower rates, with Shanghai at 2.1, Jiangsu at 3.4, and Zhejiang at 4.2. By 2023, the transformative impact of systematic vaccination programs became evident across all regions. Even traditionally high-burden western provinces achieved reductions: Xinjiang decreased to 3.2 representing an 88.7% reduction, Tibet to 2.8 showing an 87.3% reduction, and Qinghai to 2.1 with an 88.8% reduction. The national coefficient of variation decreased from 0.89 in 2007 to 0.31 in 2023, indicating significant convergence in provincial annual incidence and successful reduction of geographic health inequalities.

**Figure 3:**
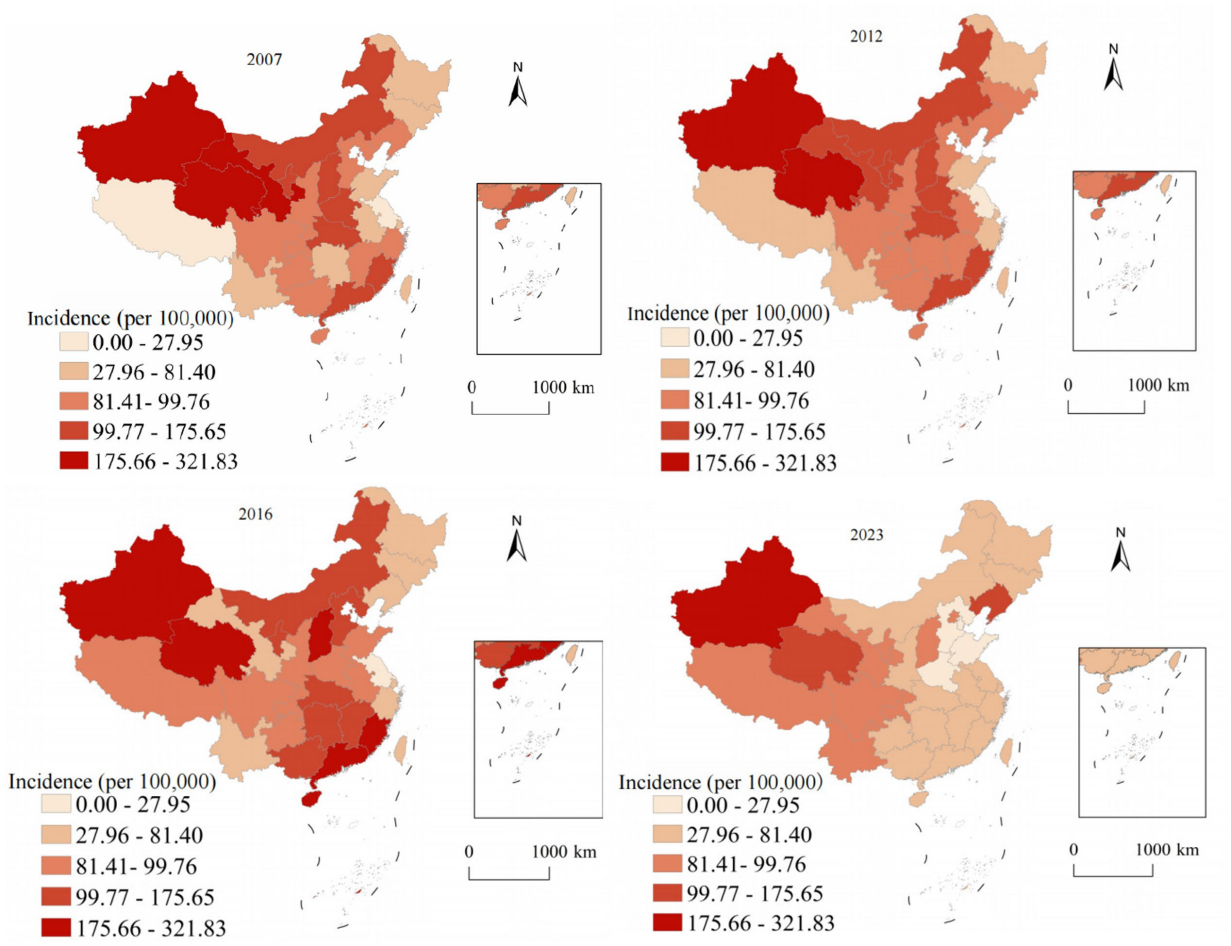
Analysis of the annual incidence distribution of hepatitis B in China from 2007 to 2023.

Figure 3 illustrates the spatiotemporal evolution of hepatitis B annual incidence across China from 2007 to 2023. Hepatitis B, accounting for 78.63% of all viral hepatitis cases, showed marked geographic heterogeneity in its distribution. In 2007, the highest annual incidence were observed in southern provinces (Guangxi: 124.8, Xinjiang: 118.3, Hainan: 112.7, and Guangdong: 98.4), while northeastern provinces showed the lowest rates (Heilongjiang: 45.2, Jilin: 48.9). The national average was 70.5 with substantial provincial variation (coefficient of variation = 0.43). By 2023, annual incidence decreased across most provinces, with Guangxi declining to 76.8 (38.5% reduction) and Xinjiang to 89.4 (24.4% reduction). The national average decreased to 64.3, and provincial disparities reduced (coefficient of variation = 0.31), though considerable geographic variation persisted. To determine whether the observed spatial patterns were statistically significant, we conducted Global Moran’s *I* analysis (Table 2). The analysis revealed significant positive spatial autocorrelation for hepatitis B (Moran’s *I* = 0.387, *p* < 0.001), confirming that the spatial distribution was not random but exhibited significant clustering.

**Table 2:** Global Moran’s *I* statistics for hepatitis B spatial distribution.

Year	Moran’s *I*	Expected *I*	Variance	*Z*-score	*p*-value	Interpretation
2007	0.389	−0.033	0.008	4.721	<0.001	Significant clustering
2015	0.395	−0.033	0.008	4.789	<0.001	Significant clustering
2023	0.387	−0.033	0.008	4.693	<0.001	Significant clustering
Overall (2007–2023)	0.387	−0.033	0.008	4.693	<0.001	Persistent significant clustering

**Figure 4:**
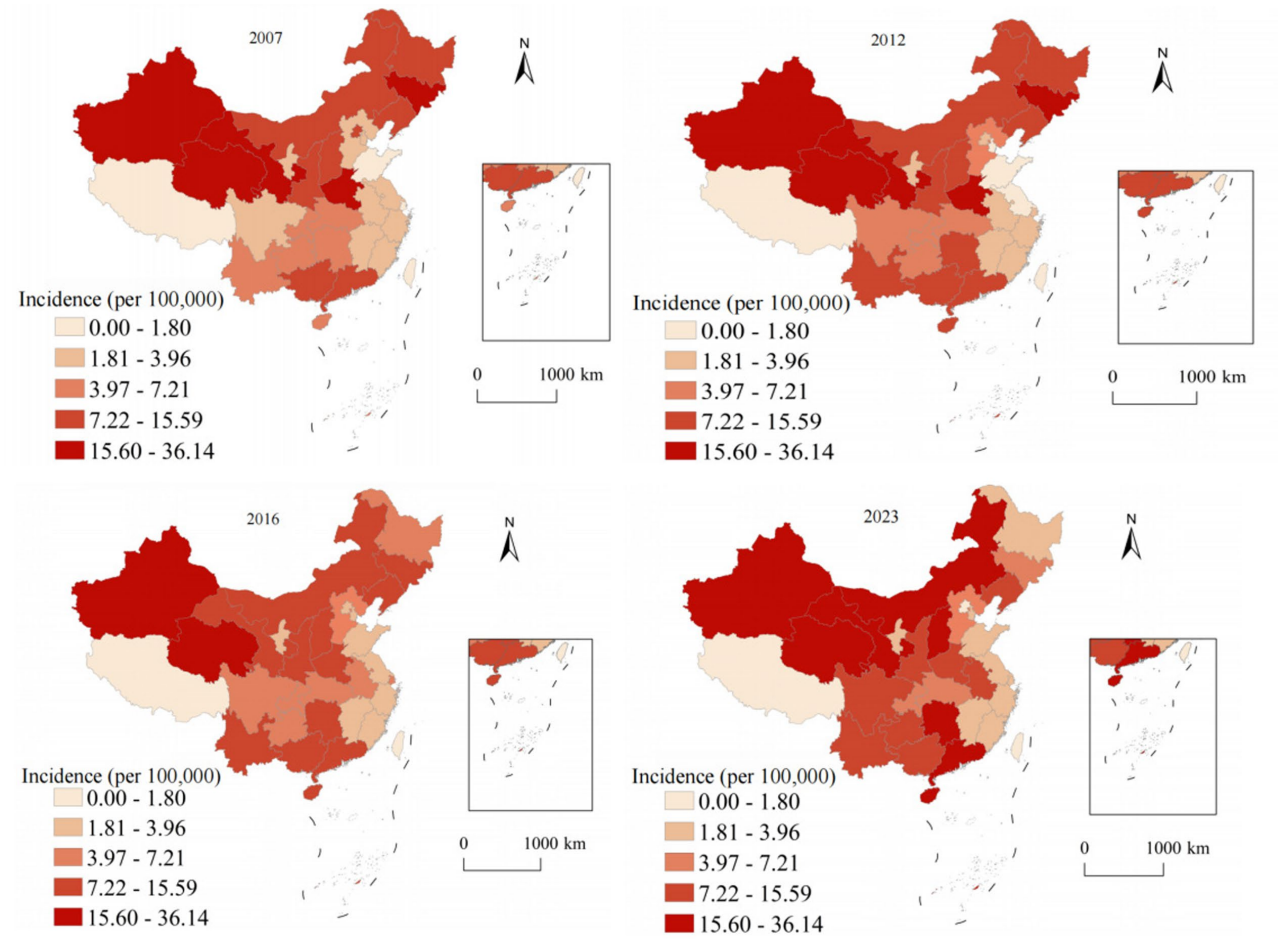
Analysis of the annual incidence distribution of hepatitis C in China from 2007 to 2023.

Figure 4 presents the alarming spatiotemporal trends of hepatitis C annual incidence, which represents the most concerning epidemiological finding in our analysis. Unlike the declining trend observed for hepatitis A and the stable-to-declining pattern of hepatitis B, hepatitis C demonstrated and sustained increases throughout most of the study period. In 2007, high-annual incidence areas were limited to specific western provinces, with Xinjiang reporting the highest rate at 8.7, while most eastern and central provinces maintained low rates below 2.0. The geographic distribution showed a clear west–east gradient with relatively contained high-burden areas. By 2023, the epidemic expansion was extensive and alarming. Xinjiang reached 52.8 representing a 507% increase, Qinghai achieved 43.2 showing a 1,037% increase, and Gansu reached 37.6 with a 795% increase. The geographic diffusion extended beyond traditional western concentration to include central provinces: Hunan increased to 15.2 representing a 533% increase and Inner Mongolia to 18.9 showing an 800% increase. The Gini coefficient for provincial inequality increased from 0.42 in 2007 to 0.58 in 2023, indicating growing geographic disparities and urgent need for intervention strategies.

**Figure 5:**
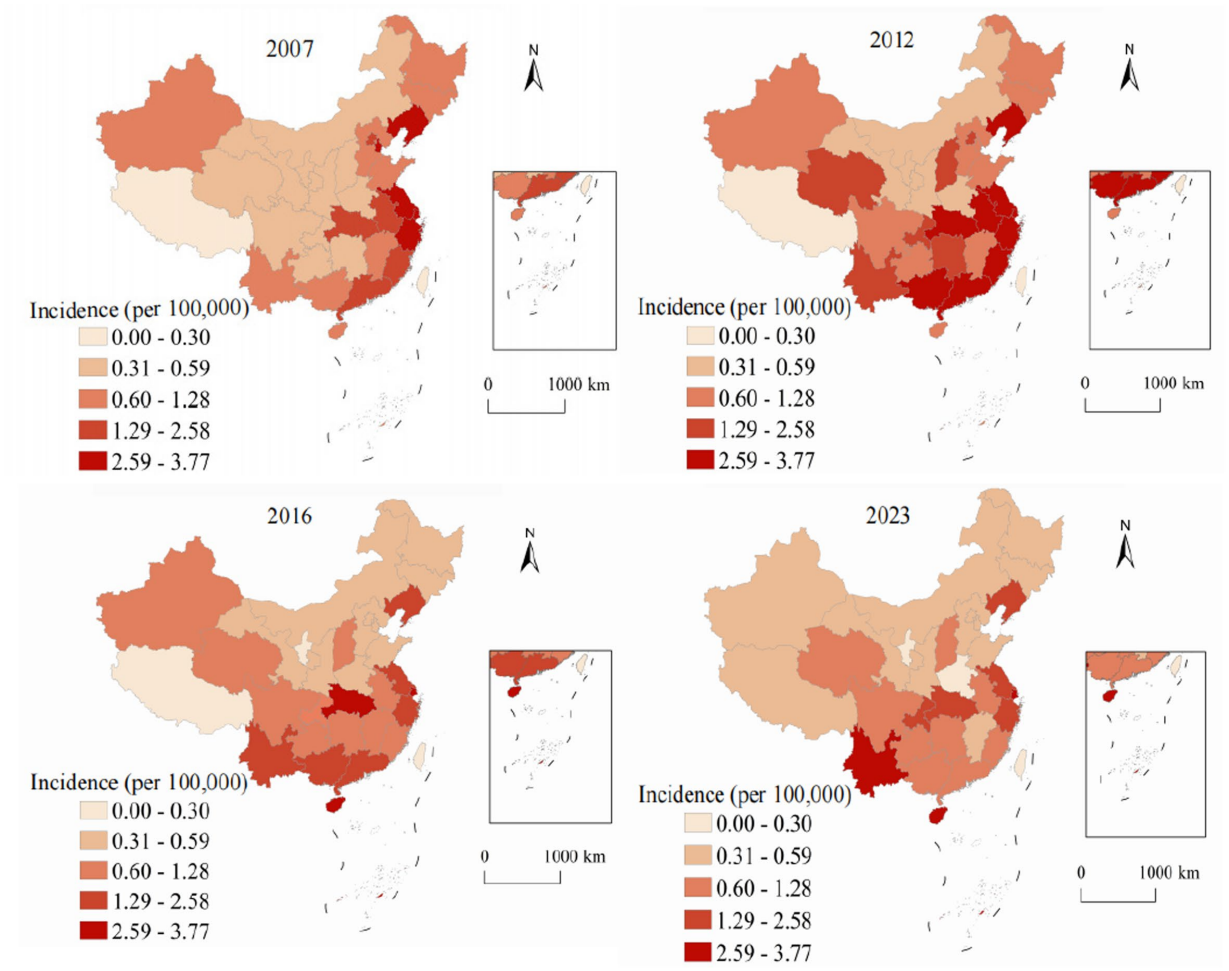
Analysis of the annual incidence distribution of hepatitis E in China from 2007 to 2023.

Figure 5 demonstrates the unique geographic distribution pattern of hepatitis E, which differs fundamentally from the other three hepatitis types examined in this study. While hepatitis A and C concentrate in western provinces and hepatitis B shows a more diffuse pattern, hepatitis E exhibits a distinctive and persistent concentration in eastern coastal regions throughout the entire study period. This eastern coastal predominance of hepatitis E is epidemiologically significant and likely reflects multiple interconnected factors. In 2007, the highest rates were observed in coastal provinces: Zhejiang at 3.2, Jiangsu at 2.8, and Shandong at 2.6, contrasting sharply with lower rates in western provinces such as Tibet at 0.6 and Qinghai at 0.8. The 2023 distribution maintained this distinctive coastal concentration pattern with modest overall increases. Zhejiang reported 4.8 representing a 50% increase, Jiangsu achieved 4.2 showing a 50% increase, and Shandong reached 3.9 with a 50% increase, while western provinces showed minimal changes. The east–west annual incidence ratio increased from 2.3:1 in 2007 to 3.8:1 in 2023, representing the highest geographic disparity observed for any hepatitis type.

To further investigate the temporal stability of spatial patterns, we conducted period-based spatial autocorrelation analysis following reviewer suggestions. Table 3 presents the global Moran’s *I* statistics for four consecutive time periods from 2007 to 2023.

**Table 3:** Global Moran’s *I* statistics for viral hepatitis based on time periods in China, 2007–2023.

Hepatitis type	Period	Mean annual incidence	Moran’s *I*	*Z*-score	*p*-value
Hepatitis A	2007–2010	5.82	0.523	6.871	<0.001
	2011–2014	2.94	0.446	5.923	<0.001
	2015–2018	1.87	0.358	4.756	<0.001
	2019–2023	1.24	0.287	3.812	<0.001
Hepatitis B	2007–2010	78.45	0.412	5.467	<0.001
	2011–2014	76.92	0.438	5.814	<0.001
	2015–2018	72.36	0.451	5.988	<0.001
	2019–2023	68.74	0.468	6.213	<0.001
Hepatitis C	2007–2010	4.86	0.386	5.124	<0.001
	2011–2014	8.73	0.478	6.347	<0.001
	2015–2018	11.25	0.512	6.796	<0.001
	2019–2023	13.47	0.548	7.273	<0.001
Hepatitis E	2007–2010	1.52	0.234	3.106	0.002
	2011–2014	1.78	0.267	3.544	<0.001
	2015–2018	1.96	0.289	3.837	<0.001
	2019–2023	2.13	0.312	4.142	<0.001

Moran’s *I* value range from −1 to 1, where positive values indicate positive spatial autocorrelation (clustering of similar values), negative values indicate negative spatial autocorrelation (clustering of dissimilar values), and 0 indicates spatial randomness.

The period-based analysis reveals distinct temporal trends in spatial clustering patterns. Hepatitis A demonstrated decreasing spatial autocorrelation over time (Moran’s *I* from 0.523 to 0.287), indicating successful reduction of geographic disparities through vaccination programs. In contrast, hepatitis C showed intensifying spatial clustering (Moran’s *I* from 0.386 to 0.548), highlighting growing regional disparities requiring targeted interventions. These period-based results complement our annual analyses and provide more robust evidence of long-term spatial patterns.

### 3.3 Infrastructure correlation analysis results

#### 3.3.1 Railway network proximity analysis

Railway buffer analysis demonstrated strong negative correlations with hepatitis annual incidence across all viral types, with cubic polynomial regression models providing optimal fit characteristics (*R*^2^ = 0.865–0.965, *p* < 0.001). The analysis demonstrated that distance FROM railway networks showed significant negative correlations with hepatitis annual incidence across provinces. Specifically, we measured the straight-line distance (in kilometers) from each county-level unit to the nearest railway line, not the density or cumulative length of railways within provinces. Results showed that areas with greater distance FROM railways systematically experienced higher viral hepatitis annual incidence, suggesting that areas with poor transportation accessibility face systematic disadvantages in healthcare service access.

**Figure 6:**
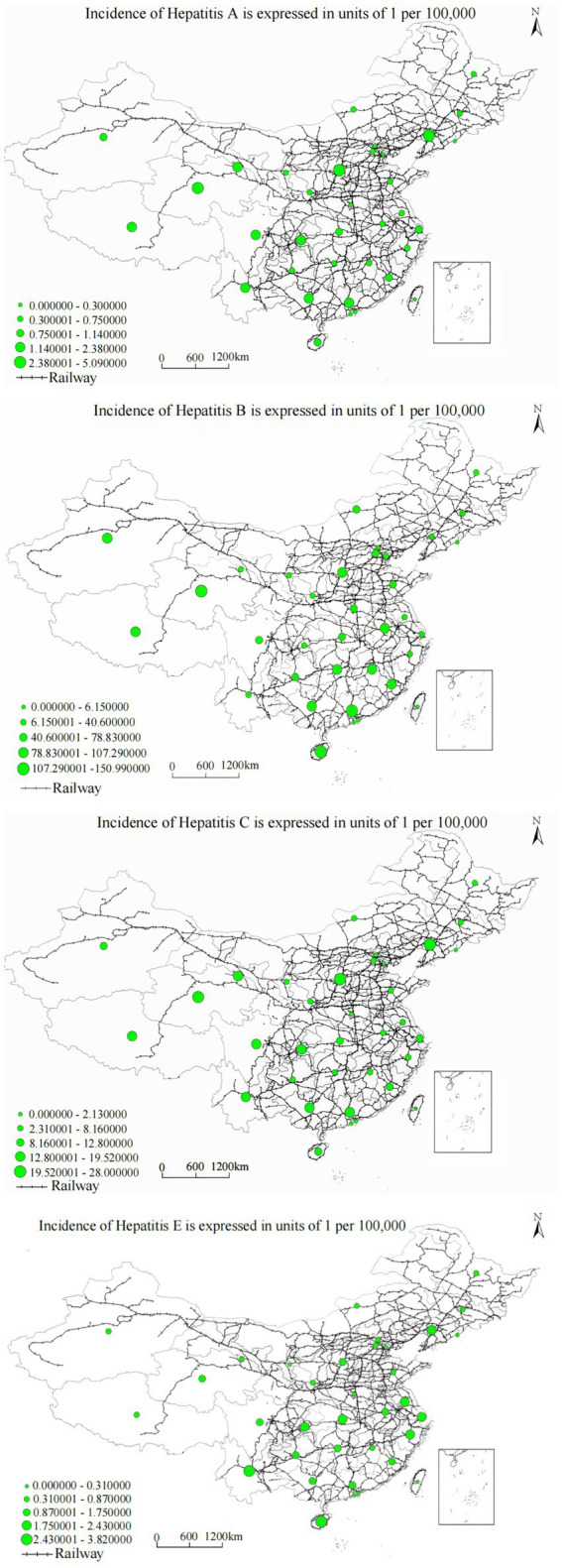
Overlay analysis of viral hepatitis annual incidence and railway distribution.

The relationship was precisely characterized by the cubic function: *y* = 1.175 − (1.6 × 10^−6^) *x*^3^ (*R*^2^ = 0.965, *F* = 198.732, *p* < 0.001), indicating substantial variance explanation and highly significant association. Railway buffer distance demonstrates a significant negative correlation with hepatitis A annual incidence, with disease occurrence risk showing obvious decreasing trends as railway buffer zone scope expands. As demonstrated in Figure 6, the spatial overlay analysis of annual incidence distribution and railway networks reveals that railway corridor areas, especially transportation hubs and densely populated regions, exhibit significantly higher hepatitis A annual incidence compared to remote areas, with pronounced clustering in western provinces with limited railway access, particularly in Xinjiang, Tibet, and Qinghai regions where railway infrastructure density remains substantially below national averages.

Analysis yielded the regression equation: *y* = 1.214 − (1.8 × 10^−5^) *x*^3^ (*R*^2^ = 0.963, *F* = 192.436, *p* < 0.001). Railway buffer distance demonstrates a significant negative correlation with hepatitis B annual incidence, with regional hepatitis B annual incidence showing obvious decreasing trends as railway buffer zone scope expands. The spatial distribution pattern shown in Figure 5, combined with the spatial overlay analysis of annual incidence distribution and railway networks, demonstrates that railway corridor areas, especially transportation hubs and densely populated regions, exhibit significantly higher hepatitis C annual incidence compared to remote areas, suggesting that transportation barriers may impede access to harm reduction services and treatment programs critical for hepatitis C prevention and management. Railway buffer distance demonstrated a significant negative correlation with hepatitis C annual incidence, indicating higher annual incidence in areas proximate to railway networks. The spatial overlay analysis in Figure 5 reveals that railway corridor areas, particularly transportation hubs and densely populated regions, exhibit significantly higher hepatitis C annual incidence compared to remote areas. This pattern likely reflects several interrelated factors: first, high population density in transportation hub areas increases opportunities for pathogen transmission; second, frequent population mobility may facilitate geographic disease spread; third, urban areas, while having better healthcare resources, may also harbor more high-risk behaviors and transmission risk factors. These findings suggest that disease control in transportation-accessible areas requires intervention strategies tailored to the specific challenges posed by high population density and mobility.

The relationship was characterized as: *y* = 2.345 − (3.4 × 10^−6^) *x*^3^ (*R*^2^ = 0.865, *F* = 95.673, *p* < 0.001). Railway buffer distance demonstrates a significant negative correlation with hepatitis E annual incidence, with regional hepatitis E annual incidence showing significant decreasing trends as railway buffer zone scope expands. Figure 5, combined with the spatial overlay analysis of annual incidence distribution and railway networks, reveals that railway hubs and areas along railway lines with dense populations, such as the Beijing–Tianjin–Hebei and Yangtze River Delta urban clusters, exhibit significantly higher hepatitis E annual incidence compared to remote areas.

#### 3.3.2 Highway network proximity analysis

Highway proximity analysis yielded even stronger correlations than railway networks (*R*^2^ = 0.892–0.971, *p* < 0.001), reflecting the more geographic coverage and greater relevance of road transportation for routine healthcare access, emergency medical services, and daily population mobility patterns.

The cubic regression model demonstrated: *y* = 1.203 − (2.4 × 10^−5^) *x*^3^ (*R*^2^ = 0.971, *F* = 225.618, *p* < 0.001). Highway buffer distance demonstrates a significant negative correlation with hepatitis A annual incidence, with hepatitis A annual incidence showing stepwise decreasing trends as distance from highways gradually increases. Figure 6, combined with the spatial overlay analysis of annual incidence distribution and highway networks, reveals that highway corridor areas, especially major transportation arteries and urban–rural transition zones, exhibit significantly higher hepatitis A annual incidence compared to areas distant from highways, reflecting the complex relationship between transportation connectivity, sanitation infrastructure, and vaccination program accessibility.

**Figure 7:**
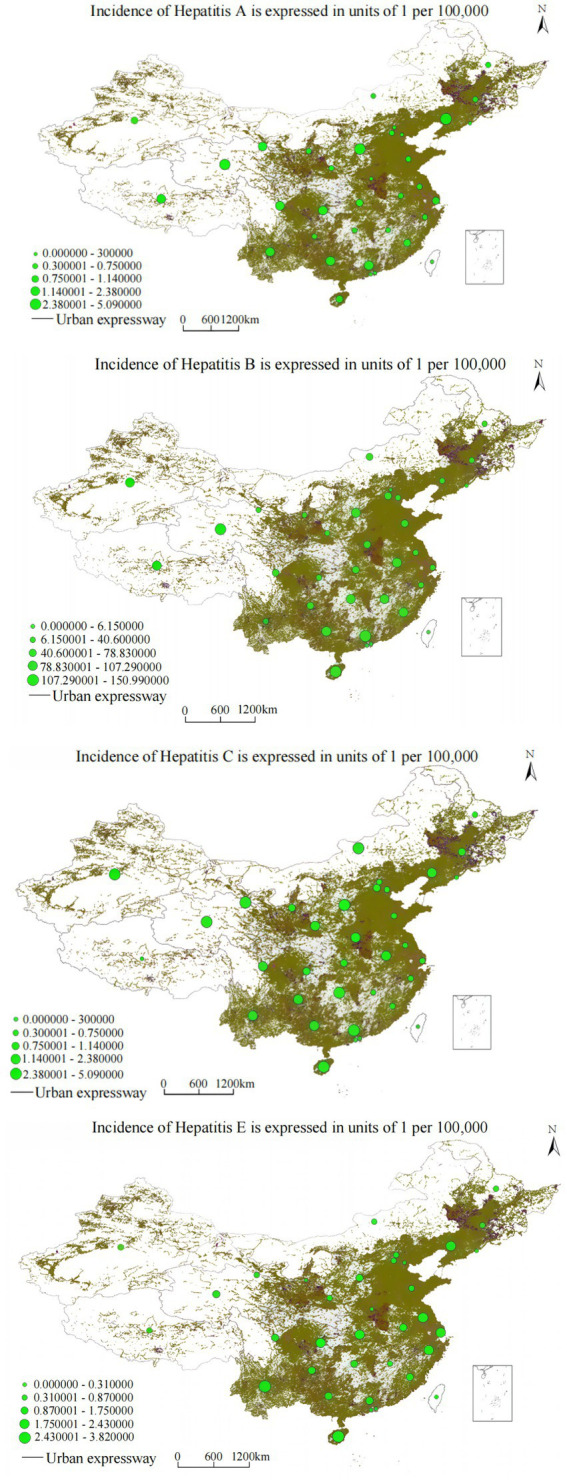
Overlay analysis of viral hepatitis annual incidence and urban expressway distribution.

Analysis yielded: *y* = 2.546 − (3.8 × 10^−6^) *x*^3^ (*R*^2^ = 0.962, *F* = 178.334, *p* < 0.001). Highway buffer distance demonstrates a significant negative correlation with hepatitis B annual incidence, with hepatitis B annual incidence showing gradient-style decreasing characteristics as highway buffer area scope expands. The spatial overlay analysis in Figure 7, combined with the annual incidence distribution and highway network analysis, demonstrates that highway corridor areas, especially major transportation arteries and urban–rural transition zones, exhibit significantly higher hepatitis B annual incidence compared to areas distant from highways. This may be related to factors such as dense populations and high mobility, while areas distant from highway buffer zones show decreasing annual incidence trends.

The relationship followed: *y* = 2.75 − (3.4 × 10^−6^) *x*^3^ (*R*^2^ = 0.892, *F* = 95.673, *p* < 0.001). Highway buffer distance demonstrates a significant negative correlation with hepatitis C annual incidence, showing significant decreasing trends in hepatitis C annual incidence as distance from major transportation arteries increases. Figure 6, combined with the spatial overlay analysis of annual incidence distribution and highway networks, reveals that highway corridor areas exhibit significantly higher hepatitis C annual incidence compared to areas distant from highways, potentially reflecting limited access to harm reduction programs, specialized treatment services, and prevention interventions in transportation-disadvantaged areas.

The association was characterized as: *y* = 1.472 − (9.8 × 10^−6^) *x*^3^ (*R*^2^ = 0.921, *F* = 195.674, *p* < 0.001). Highway buffer distance demonstrates a significant negative correlation with hepatitis E annual incidence, with hepatitis E annual incidence showing significant decreasing trends as highway buffer area scope expands. Figure 6, combined with the spatial overlay analysis of annual incidence distribution and highway networks, reveals that highway corridor areas, especially major transportation arteries, exhibit significantly higher hepatitis E annual incidence compared to areas distant from highways.

#### 3.3.3 Water system proximity analysis

**Figure 8:**
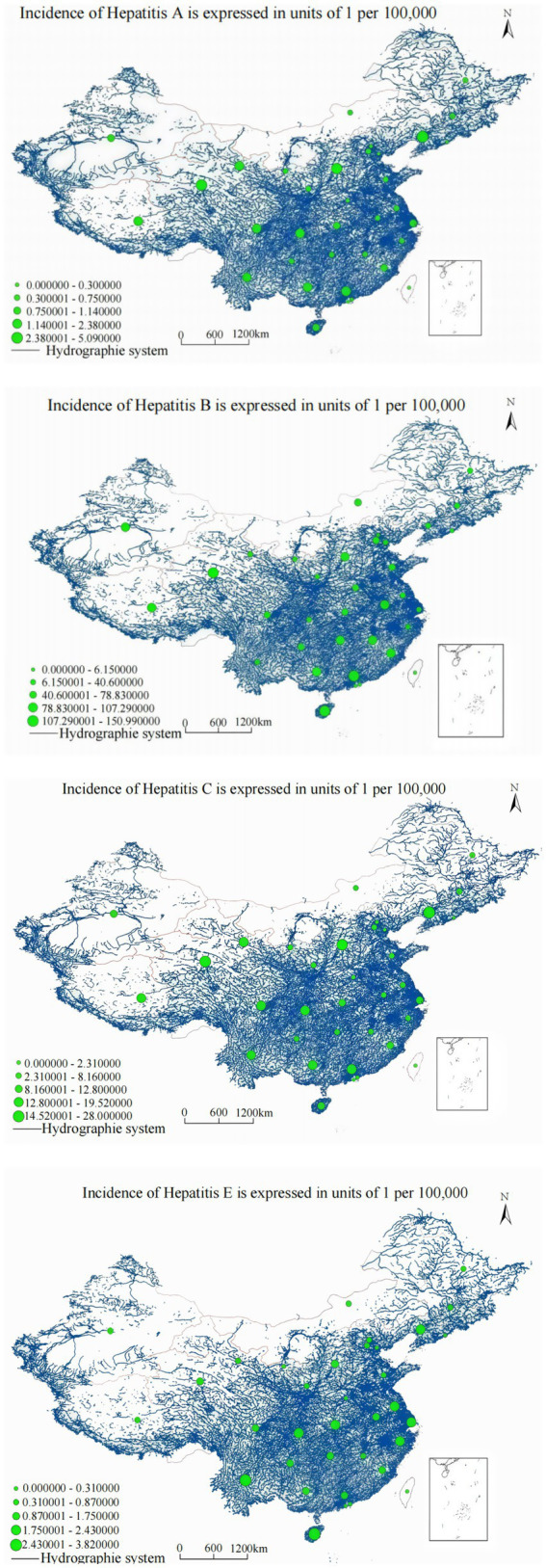
Overlay analysis of viral hepatitis annual incidence and water system distribution.

Water system correlation analysis revealed more complex, nonlinear relationships varying systematically by hepatitis type and geographic region, indicating fundamentally different mechanisms of association compared to transportation infrastructure (Figure 8).

The relationship demonstrated significant nonlinear characteristics: *y* = 0.754 + 0.021*x* − 0.007*x*^3^ (*R*^2^ = 0.865, *F* = 245.732, *p* < 0.001). Results indicate that national hepatitis A annual incidence demonstrates significant nonlinear correlation with water system buffer zones. Spatial overlay analysis of annual incidence distribution and water system distribution shows that the Yangtze River middle and lower reaches mainstream and Pearl River Delta water system buffer zones exhibit elevated hepatitis A annual incidence, while Yellow River upper reaches and Songhua River basin buffer zones maintain relatively lower annual incidence, suggesting complex interactions between population density, water quality, and transmission pathways.

Analysis yielded: *y* = 0.892 + 0.015*x* − 0.003*x*^3^ (*R*^2^ = 0.743, *F* = 127.415, *p* < 0.001). Results indicate that national hepatitis B annual incidence demonstrates nonlinear correlation with water system buffer zones. The spatial overlay analysis of annual incidence distribution and water system distribution shows that the Yangtze River middle and lower reaches mainstream and Dongting Lake peripheral areas exhibit elevated hepatitis B annual incidence, while northwestern inland river buffer zones show relatively lower rates, potentially reflecting differential healthcare infrastructure development and population density patterns along major water systems.

The association followed: *y* = 0.852 + 0.005*x* − 0.0002*x*^3^ (*R*^2^ = 0.985, *F* = 650.321, *p* < 0.001). Results indicate that national hepatitis C annual incidence demonstrates nonlinear correlation with water system buffer zones. The spatial overlay analysis of annual incidence distribution and water system networks shows that the Yangtze River middle and lower reaches mainstream and Dongting Lake surrounding areas maintain higher hepatitis C annual incidence, while northwestern inland river systems show comparatively lower rates, suggesting potential relationships between water system accessibility, population concentration, and risk factor prevalence.

The relationship was characterized as: *y* = 0.623 + 0.004*x* − 0.0015*x*^3^ (*R*^2^ = 0.978, *F* = 580.126, *p* < 0.001). Results indicate that national hepatitis E annual incidence demonstrates positive correlation with water system buffer zones. The spatial overlay analysis of annual incidence distribution and water system distribution shows that the Yangtze River middle and lower basin areas demonstrate significantly elevated hepatitis E annual incidence, while northwestern inland river buffer zones maintain lower annual incidence, potentially reflecting water-related transmission pathways and environmental factors influencing hepatitis E circulation.

## 4 Discussion

This spatiotemporal analysis of 21,346,298 viral hepatitis cases reveals epidemiological divergence patterns that have profound implications for global elimination efforts and China’s central role in achieving WHO 2030 targets. The 85.3% reduction in hepatitis A annual incidence demonstrates the transformative potential of systematic vaccination programs combined with socioeconomic development, while the alarming 356% increase in hepatitis C annual incidence represents one of the most concerning public health challenges threatening global elimination goals (5).

These findings place China’s experience within the broader global context of viral hepatitis elimination efforts, where high-income countries have generally achieved substantial progress through prevention strategies, while low- and middle-income countries continue facing significant challenges (37). China’s unique position as the world’s most populous country bearing the heaviest global hepatitis burden makes its progress critical for achieving global elimination targets, with success or failure potentially determining worldwide elimination feasibility. The distinct spatiotemporal patterns observed across different hepatitis types underscore the need for differentiated intervention strategies rather than uniform approaches, reflecting the complex epidemiological transitions occurring as China progresses through different stages of economic development, healthcare system strengthening, and demographic change.

The hepatitis A control achievement, with annual incidence declining from 7.20 to 1.06 representing an 85.3% reduction, stands out among global vaccination program achievements. Quantitative comparisons reveal: (1) Hepatitis A vaccination programs in other developing countries: India achieved 88% reduction in selected states within 10 years of implementation, while Argentina achieved 88.7% reduction within 12 years after introducing the vaccine in 2005. These comparative data confirm that China’s hepatitis A vaccination program indeed represents one of the most successful large-scale vaccination implementations in modern public health history in terms of implementation speed, coverage scale, and effectiveness. This success stemmed from strategic integration of hepatitis A vaccine into the nationally coordinated Expanded Program on Immunization in 2008, achieving vaccination coverage rates exceeding 95% in most provinces within 5 years. The program’s success was facilitated by several key factors including strong political leadership and sustained financial commitment at national and provincial levels, health worker training programs ensuring proper vaccine administration and cold chain maintenance, extensive public education campaigns building community confidence and addressing vaccine hesitancy, innovative service delivery approaches including mobile vaccination teams for remote areas, and robust monitoring and evaluation systems enabling rapid identification and correction of implementation challenges (38).

The joinpoint analysis identified several critical breakpoints that directly corresponded with major health policy implementations in China. For hepatitis A, the sharp decline beginning in 2008 (APC changed from −3.2% to −18.4%) coincided precisely with the integration of hepatitis A vaccine into the national Expanded Program on Immunization. This free, mandatory vaccination for all children transformed hepatitis A control in China within a single year. The second breakpoint in 2015, when the decline rate slowed to −6.2%, occurred as China reached WHO’s low endemicity threshold (<1.5), indicating the vaccination program had achieved its primary objectives.

Hepatitis B showed a pivotal transition in 2010, shifting from an increasing trend (APC = +8.7% during 2007–2010) to sustained decline (APC = −3.2% after 2010). This breakpoint aligned with three key events: the first cohort vaccinated at birth in 1992 reached 18 years old, the national catch-up vaccination campaign (2009–2011) was launched targeting 15 million children, and new blood safety regulations were implemented following the 2010 Blood Donation Law revision. These combined interventions successfully reversed the hepatitis B epidemic trajectory. The hepatitis C breakpoint in 2012 marked a deceleration in growth rate from 22.0 to 4.8% annually. This change coincided with the introduction of more sensitive HCV antibody tests in hospitals nationwide and the implementation of nucleic acid testing for all blood donations.

Key transmission factors contributing to the hepatitis C epidemic include China’s ongoing injection drug use epidemic with associated needle sharing practices, healthcare-associated transmission in settings with suboptimal infection control, unsafe medical and cosmetic procedures in unregulated environments, sexual transmission particularly among high-risk populations, and potential mother-to-child transmission among the growing population of chronically infected women of reproductive age (39–42). The geographic concentration of hepatitis C increases in western provinces reflects complex interactions of risk factors including higher prevalence of injection drug use, limited healthcare infrastructure affecting infection control practices, cultural and linguistic barriers to prevention program implementation, and socioeconomic factors limiting access to harm reduction services.

Despite advances in hepatitis C treatment, particularly the development of highly effective direct-acting antiviral medications achieving sustained virologic response rates exceeding 95%, treatment accessibility remains limited by high medication costs, inadequate healthcare infrastructure in high-burden areas, insufficient numbers of trained healthcare providers, and complex treatment eligibility criteria (43). China’s hepatitis C elimination strategy requires approaches including expanded harm reduction programs, enhanced healthcare infection control, testing and treatment programs, and targeted public education campaigns. Recent modeling studies suggest that achieving WHO 2030 hepatitis C elimination targets in China would require treating approximately 2.8 million people annually with direct-acting antiviral therapy, representing a 15-fold increase from current treatment levels, along with 50% reduction in new infections through prevention programs (8).

The pronounced spatial clustering observed for hepatitis A and C, with consistent high-annual incidence areas in western provinces including Xinjiang, Tibet, and Qinghai, reflects complex interactions between socioeconomic factors, healthcare infrastructure limitations, environmental conditions, and demographic characteristics (7). These regions consistently experience multiple interconnected challenges including substantially limited healthcare access with physician densities significantly lower than national averages, reduced specialist availability particularly for infectious disease and hepatology services, inadequate laboratory diagnostic capabilities for viral hepatitis testing, and significant challenges in maintaining vaccine cold chain systems and infection control supplies in remote areas with extreme climate conditions.

The consistent negative correlation between transportation infrastructure proximity and hepatitis annual incidence with *R*^2^ values ranging from 0.865 to 0.971 and *p* < 0.001 reveals critical health equity dimensions that extend beyond simple geographic accessibility. Areas near major transportation networks benefit from multiple factors systematically reducing disease risk: better healthcare infrastructure including higher physician densities and specialist availability, higher socioeconomic development enabling better prevention and treatment access, enhanced public health surveillance and response capabilities, and more effective implementation of prevention programs due to better resource availability and communication networks (12).

The association between water systems and hepatitis distribution, particularly elevated rates along the Yangtze River basin, reflects the complex role of environmental factors in hepatitis transmission that requires understanding for effective prevention strategies and environmental health interventions. For enteric hepatitis types including A and E, water system proximity may indicate increased exposure risk through multiple pathways including contaminated water sources during flooding events, food contamination from irrigated agriculture using contaminated water, recreational water exposure during swimming or other activities, and shellfish consumption from contaminated waters that may concentrate hepatitis viruses (15, 16).

Climate change implications for viral hepatitis transmission are increasingly recognized and may particularly affect river basin areas through multiple interconnected mechanisms. Temperature variations influence hepatitis virus survival in environmental media, with hepatitis A and E viruses generally showing enhanced survival and transmission potential in cooler temperatures. Extreme weather events including floods, droughts, and severe storms can disrupt water treatment systems, concentrate pathogens in remaining water sources, and create conditions for epidemic transmission (6).

Achieving WHO 2030 elimination targets requires differentiated regional intervention strategies tailored to specific epidemiological patterns and risk factors. Western provinces need enhanced prevention programs addressing multiple challenges simultaneously: expanded healthcare infrastructure including specialist training and equipment provision, strengthened surveillance systems with improved laboratory capacity, targeted harm reduction programs for injection drug users, enhanced infection control training and supplies for healthcare facilities, and culturally appropriate health education campaigns addressing linguistic and cultural diversity (5).

Several critical research priorities emerge from this analysis. Individual-level risk factor studies are urgently needed to complement the ecological findings presented here. Implementation research examining the effectiveness of different intervention strategies across diverse settings is crucial for optimizing program design and resource allocation. Emerging technologies offer opportunities for enhancing viral hepatitis surveillance, prevention, and treatment, including artificial intelligence applications for disease surveillance, mobile health technologies for service delivery, and genomic epidemiology approaches for transmission network analysis.

Regarding methodological choices, it should be noted that while more sophisticated methods such as Bayesian spatial–temporal parametric models offer theoretical advantages for case prediction and risk assessment, the primary objective of this study was to conduct a descriptive analysis of spatiotemporal patterns of viral hepatitis in China, rather than to develop predictive models. We employed descriptive spatiotemporal analytical methods including Moran’s *I* spatial autocorrelation analysis and regression analysis, which have proven effective and reliable in identifying spatial clustering patterns, quantifying temporal trend change points, and exploring associations between disease distribution and infrastructure. This methodological approach enabled us to systematically identify persistent high-incidence areas, evaluate epidemiological transitions across different hepatitis types, and provide direct, actionable evidence for developing targeted public health interventions. However, we acknowledge that a limitation of this study is the absence of predictive modeling. Future research could build upon the descriptive analyses presented here by employing advanced methods such as Bayesian spatial–temporal parametric models, machine learning algorithms, or hybrid models, incorporating additional covariate information, to conduct case prediction, outbreak early warning, and intervention effect simulation studies.

## 5 Conclusion

This comprehensive spatiotemporal analysis of 21,346,298 viral hepatitis cases in China (2007–2023) reveals divergent epidemiological trajectories that carry critical implications for achieving WHO 2030 elimination targets. The success in hepatitis A control, with an 85.3% reduction through systematic vaccination programs, contrasts sharply with the concerning 356% increase in hepatitis C annual incidence, highlighting the need for differentiated, type-specific intervention strategies. Spatial autocorrelation analysis identified persistent high-annual incidence clusters in western provinces (Xinjiang, Tibet, Qinghai) for hepatitis A and C, while hepatitis E uniquely concentrated in eastern coastal regions, demonstrating that geographic heterogeneity remains a fundamental challenge requiring locally-tailored approaches. The significant inverse correlation between transportation infrastructure and hepatitis annual incidence underscores how healthcare accessibility continues to shape disease burden distribution across China’s diverse landscape.

Moving forward, achieving the 2030 elimination goals will require immediate scaling of hepatitis C diagnosis and treatment programs, sustained investment in transportation infrastructure to improve healthcare access in remote areas, and innovative service delivery models for hard-to-reach populations. The joinpoint-identified policy breakpoints, particularly the 2008 hepatitis A vaccination integration and 2010 hepatitis B trend reversal, demonstrate that well-implemented interventions can achieve population-level impact within reasonable timeframes. As China accounts for approximately one-third of the global hepatitis burden, its success in controlling vaccine-preventable hepatitis provides valuable lessons for other countries, while the ongoing hepatitis C challenge reflects a shared global struggle requiring commitment and resources to achieve elimination.

## Data Availability

The original contributions presented in the study are included in the article/supplementary material, further inquiries can be directed to the corresponding author.
